# Identification of Possible Pre-Slaughter Indicators to Predict Stress and Meat Quality: A Study on Heavy Pigs

**DOI:** 10.3390/ani10060945

**Published:** 2020-05-29

**Authors:** Luca Sardi, Alessandro Gastaldo, Marzia Borciani, Andrea Bertolini, Valeria Musi, Giovanna Martelli, Damiano Cavallini, Giulia Rubini, Eleonora Nannoni

**Affiliations:** 1 Department of Veterinary Medical Sciences, University of Bologna, Via Tolara di Sopra 50, 40064 Ozzano Emilia (BO), Italy; luca.sardi@unibo.it (L.S.); damiano.cavallini@unibo.it (D.C.); giulia.rubini8@unibo.it (G.R.); eleonora.nannoni2@unibo.it (E.N.); 2 Foundation C.R.P.A. (Research Centre on Animal Production) Studies and Researches, Viale Timavo 43/2, 42121 Reggio Emilia, Italy; a.gastaldo@crpa.it (A.G.); m.borciani@crpa.it (M.B.); a.bertolini@crpa.it (A.B.); v.musi@crpa.it (V.M.)

**Keywords:** animal welfare, transport, stress, pigs, blood parameters, meat quality, indicators

## Abstract

**Simple Summary:**

This study aimed at identifying, on the basis of objective evaluations (blood parameters): (1) the pre-slaughter treatments that result in higher stress for heavy pigs and can therefore be used as pre-slaughter stress indicators; (2) the meat quality parameters that show the largest variation as a response to the stress experienced during pre-slaughter handling. Two blood parameters (cortisol and creatine kinase) were used to categorize pigs into two groups (clusters) depending on transport conditions: “higher stress” and “lower stress”. Our results indicate that the variables/indexes which differed more widely between the two clusters (namely, average vehicle speed during transport, welfare slaughter score, overall transport and slaughter welfare index (TSWI), distance travelled, group stability, and behaviors—slips, falls, and overlaps—during unloading) might be considered as the best descriptors of the welfare conditions experienced by Italian heavy pigs during pre-slaughter handling. However, we observed no consistent effects of the stress experienced during pre-slaughter handling on meat quality, which warrants the need for further studies addressing: (1) the individual stress response and meat quality variability in pigs within the same transport; (2) the relationships between the variables examined and meat quality, with the aim to improve the TSWI.

**Abstract:**

This study aimed at identifying possible pre-slaughter indicators and/or indexes to be used to predict pig stress response and meat quality variation. Data were collected on 44 shipments (loads) of Italian heavy pigs. For each shipment, several pre-slaughter parameters were recorded on farm, during transport, and at the slaughterhouse. Blood and meat samples were taken from 10 pigs from every of the 44 loads included in the study (N = 440). Blood samples were used to assess cortisol and creatine kinase levels, whereas meat samples were used to assess meat quality (pH, instrumental color, tenderness, water-holding capacity, and sensory analysis). Cluster analysis of blood parameters allowed the categorization of the shipments into two main clusters: Lower Stress (LS) and Higher Stress (HS). The variables/indexes statistically differing between the two clusters were: average vehicle speed during transport, welfare index at slaughter (i.e., “slaughter score”), overall transport and slaughter welfare index (TSWI), distance travelled, and behaviors (slips, falls, overlaps) during unloading, which appeared to be the best descriptors of the welfare conditions experienced by Italian heavy pigs during pre-slaughter handling. No consistent effects of the stress level experienced on meat quality was detected, which warrants the need for further studies conducted under more variable pre-slaughter conditions, with the aim of simplifying and improving the TSWI.

## 1. Introduction

A detailed study of the associations between pre-slaughter handling and stress response would benefit the industry and animals by reducing transport losses and promoting good animal welfare and meat quality [1]. In the present work, we focused on the welfare during pre-slaughter handling, of Italian heavy pigs, which are transported and slaughtered at a minimum age of 9 months and at the average body weight (BW) of 160 kg ± 10% [2].

Pre-slaughter handling “consists of several stages, starting when pigs leave their pen and including transport, lairage, stunning, and exsanguination” [3]. All these practices can induce stress, a term which will be used in the present work with the meaning of “a way of suggesting negative implications (defined as acute or chronic stress) on pig welfare” during pre-slaughter handling and transport [1]. Overall, the stress experienced by pigs in these phases is the result of complex interactions between on-farm conditions (e.g., housing system, feed withdrawal, and handling) and conditions experienced during transport (e.g., vehicle design, transport duration, space allowance, climate) and lairage (e.g., duration and handling), which affect animal losses, pigs’ physiological and behavioral responses before slaughter, and carcass and meat quality variation [4].

The physiological response to transport has been studied considering various blood parameters. In particular, although its values vary widely across the literature and comparisons among studies are not always easy to make, cortisol has been frequently used as an indicator of acute stress resulting from handling, transport, and restraint [5]. Creatine Kinase (CK) in blood increases when animals are subjected to intense physical activity (as that experienced during loading or harsh handling) or muscle damage; therefore this enzyme can be used, together with other parameters, as a long-term indicator of welfare during transport [6], since its concentration peaks at 6 h and returns to basal levels between 8 and 48 h after muscle damage [7,8]. Blood CK increases during transport (it is released in the bloodstream due to the rupture of muscle cell membranes caused by vigorous muscle effort) and decreases during lairage [9]. Although less common in the literature, another possible stress indicator is blood aldolase. Similarly to CK, aldolase is an enzyme which leaks into the circulation from damaged muscle, and its values increase over time (peaking after at least 48–72 h) [10]. A study showed that aldolase activity is correlated to meat quality traits, such as pH and sarcoplasmic and myofibrillar protein solubility [11], all indicators of poor water-holding capacity. Furthermore, in human medicine, aldolase has been indicated as a more accurate and objective indicator of muscle damage than CK due to its lower inter-individual variability [10]. For these reasons, aldolase was also assessed in the present work as a possible, long-term muscle damage indicator.

Several studies investigated the correlations between blood stress parameters (and other physiological indices) and meat quality, but their results were inconsistent. Additionally, the correlations observed ranged from weak to moderate, indicating that blood stress parameters can only be used as a complementary measurement in the assessment of pigs’ response to transport stress [12,13,14].

A recent review also highlighted that the current literature available on pig transportation mostly focuses on pigs at the average market weight (100–135 kg) [1]. Therefore, there is a considerable lack of knowledge regarding the handling of smaller and larger pigs, which may react differently to pre-slaughter stressors. Lighter and heavier pigs are more susceptible to heat stress, especially during transport of long (between 8 and 24 h [1]) and short duration (90 min [15]), respectively.

Our broad aim was identifying (and possibly validating) pre-slaughter indicators and animal welfare indexes—such as the transport and slaughter welfare index (TSWI) to be used to predict stress response and meat quality variation in heavy pigs. More specifically, the aim of this work was two-fold: (1) to assess, based on objective measures (blood parameters), the impact of pre-slaughter events (loading, transport, unloading, lairage, and stunning), as single factors or in combination, on heavy pigs’ stress response; (2) to identify the meat quality parameters that show the largest variation in response to variations in blood parameters.

## 2. Materials and Methods

### 2.1. Ethical Statement

The experiment was carried out in full accordance with the European legal requirements for the protection of pigs on farms (Directive 2008/120/EC [16]), during transport (Council Regulation -EC- N° 1/2005 [17]), and at slaughter (Council Regulation -EC- N° 1099/2009 [18]). Animals were raised in commercial farms, transported and sacrificed for human meat consumption in the respect of the above-mentioned legislation. Pigs were not subjected to any experimental invasive procedure in vivo (blood samples were taken at exsanguination once the animals were stunned, and meat samples were taken from the carcasses). For these reasons, these trials did not fall within the field of application of the Directive 2010/63/EU [19] on the protection of animals used for scientific purposes and therefore did not require a specific authorization by the local animal welfare and ethical review body.

### 2.2. Experimental Design and Sampling Scheme

Data collection was carried out across one year on 44 shipments of Italian heavy pigs. A shipment was defined in the present study as “a group of animals undergoing the same pre-slaughter handling (originating from the same farm, transported at the same time on the same truck, and subjected to the same lairage and slaughter conditions)”. The shipments originated from 11 randomly selected, commercial farms and were made to the same commercial slaughter plant, all located in Northern Italy (Emilia Romagna region). The slaughterhouse involved in the trial has a manual, head-only electrical stunning system with a restraining cage. Each farm provided 4 loads of pigs, of which 2 transported during the warm season (April–September) and 2 transported during the cold season (October–March). The TSWI was assessed for every shipment (i.e., load) of pigs (N = 44). This work represents the first description and the first ever attempt of validating the TSWI and its single components. The index will be described in more in detail in the Section 2.3, and additional information on the methodology for score assessment and index calculation is provided in the attached Supplementary Materials.

### 2.3. Transport and Slaughter Welfare Index (TSWI)

The TSWI has been proposed by a group of researchers from the Research Centre on Animal Production (CRPA, Reggio Emilia, Italy) with the aim to create a new tool for the evaluation of farm animals’ welfare during transport. Similarly to the Farm Welfare Index (FWI) [20], the TSWI is based on the compilation of three checklists for each shipment and on a calculation system in which the different parameters assessed on departure at the farm, during transport, and at the slaughterhouse are weighed. This allows the attribution of a final score to each shipment. The evaluations start with a relatively limited number of objective parameters that can be easily measured during the farm and slaughter visits and during transport.

For each shipment, the assessor is asked to fill in 3 checklists:

- **On-departure (from the farm) checklist** (to be filled in at the farm, when animals are moved from the pen to the truck). This checklist results in an “on-departure score”, which ranges from a minimum of −30.5 points (pts) for the lowest welfare level to a maximum of 15.5 pts for the highest welfare level. The main aspects considered are:
  ▪ loading duration;
  ▪ path from the pen to the truck (length, width, design, flooring, presence of internal and external corridors, ramps, loading facilities) and time taken to move and load the pigs;
  ▪ handling (tools used and mode of use);
  ▪ pig behaviors during handling (slipping, falling, overlapping).
- **Transport checklist** (to be filled in during the journey). This checklist results in a “transport score” which ranges from a minimum of −18 pts for the lowest welfare level to a maximum of 9.5 pts for the highest welfare level. The main aspects considered are:
  ▪ distance and duration of the journey;
  ▪ space allowed to each pig;
  ▪ presence and number of drinkers;
  ▪ cooling systems;
  ▪ other characteristics of the truck (possibility to inspect animals and take care of them, internal illumination, floor type and condition, presence or absence of bedding).
- **Slaughter checklist** (to be filled in at the slaughterhouse during unloading, lairage, and stunning). This checklist results in a “slaughter score”, which ranges from a minimum of −41.5 pts for the lowest welfare level to a maximum of 50.5 pts for the highest welfare level. The main aspects considered are:
  ▪ duration of the unloading operations;
  ▪ path from the truck to the lairage pen and from the lairage pen to the stunning area (flooring, passages, presence of one-way gates);
  ▪ handling (tools used and mode of use);
  ▪ pig behaviors during handling (slipping, falling, overlapping).
  ▪ lairage pens (stocking density, ventilation, illumination, thermal insulation, conditions of floors and surfaces, type of pens, presence of mobile partitions, drinkers, cooling systems);
  ▪ stunning area (partitions, gates, devices, method of stunning, stun-to-stick interval, procedure for the use and check of the efficiency of the stunning system, emergency stunning procedures, training of the personnel involved).

All data are then submitted to the calculation system, which weights the different measures from the three checklists and calculates the overall TSWI. Additional details on how the factors in the TSWI are weighed can be found in the Supplementary Materials.

In the present study, the same two researchers (who were involved in the index formulation) worked together and collaborated in filling in the three checklists for every shipment (on farm, during transport, and at slaughter). Before the experiment, the protocol was tested by the same two assessors on some pilot shipments (from loading until stunning) to ensure the feasibility of the whole protocol and to verify the time necessary to take all measurements. The assessors also carried out several inspections at the slaughterhouse involved in the trial to ensure the applicability of the slaughter checklist.

In the present work, we chose to include only a single slaughterhouse in the data collection in order to focus on transport variables and to avoid the confounding effects of factors related to the slaughter plant. Furthermore, besides being included within the calculated index, some measures will be also elaborated and discussed separately from the index. The choice of the measures was made based on their expected importance in the overall animal welfare level during pre-slaughter handling and/or on their easy assessment under commercial conditions. The measures chosen are: on-departure (at farm) score, transport score, slaughter score, number of pigs loaded/unloaded per hour, loading and unloading duration, waiting time of the loaded truck at the farm (before leaving) and at the slaughter plant (before unloading), transport duration, total journey duration (from loading to unloading), lairage duration (from unloading to stunning), and behavior of the pigs during loading/unloading (i.e., slipping, falling, overlapping).

### 2.4. Blood Sampling and Analysis

A blood sample (10 mL) was collected at exsanguination from 10 randomly selected animals from each of the 44 loads (N = 440). Blood was collected in lithium heparin tubes and immediately stored at +4 °C to be transferred to the laboratory of the Department of Veterinary Medicine of the University of Bologna. Blood tubes were then centrifuged (at 2000× *g* for 20 min), and plasma was separated and stored at −20 °C, pending subsequent analysis for cortisol, CK, and aldolase.

Cortisol concentration (expressed as ng/mL) was then measured with a radioimmunological assay as described by Bacci et al. [21]. Cross reactions of various steroids with the rabbit anti-cortisol serum were as follows: cortisol 100%, cortisone 20.4%, 11-deoxicortisol 49.8%, corticosterone 1.13%, progesterone 0%. The mean cortisol recovery was 96.6 ± 2.2%. The intra- and inter-assay coefficients of variation (5 determinations in triplicate) were 4.30% and 7.12%, respectively. The assay sensitivity was 1.23 pg/tube.

Creatine kinase and aldolase concentrations (both expressed as U/L) were measured using two commercially available kits for their assessment in plasma (CK Nac Liquid and Aldolase, Sentinel Diagnostics, Milano, Italy), both based on a colorimetric method with subsequent spectrophotometric UV readings. The intra- and inter-assay coefficients of variation for CK (5 determinations in triplicate) were both below 13%.

### 2.5. Meat Quality and Sensory Analysis

At the slaughter plant, pH values of the longissimus thoracis and lumborum (LTL) muscle were assessed at the 2nd/3rd last rib level using a portable pH meter equipped with a temperature compensation probe (model 250A; Orion Research, Boston, MA) 45 min post-mortem. Two chops of the LTL muscle were collected from each carcass from the same anatomical location. The first chop (approximately 6 cm thick) was used for meat quality analysis, whereas the second one (approximately 12 cm thick) was used for sensory analysis. Meat quality and sensory assessments were carried out on the same animals from which the blood samples were collected (10 animals per shipment, N = 440).

For meat quality assessment, LTL muscle chops were individually stored in plastic bags, placed in a portable cooler, and immediately transferred to the laboratories of the Department of Veterinary Medical Science of the University of Bologna. Upon arrival, the samples were stored at +4 °C pending subsequent analysis, which was carried out the day after. At 24 h post-mortem, a thin layer of the LTL muscle chop surface was removed to expose the muscle and measure color, pH, drip loss, cooking loss, and shear force. After a 30 min blooming time (during which the sample was wrapped in an oxygen-permeable plastic film), the instrumental color of the LTL muscle samples was assessed by means of a Minolta Chromameter CR-400 (Konica Minolta optics INC., Tokyo, Japan) set with the D65 illuminant, according to the CIE Lab (L*, a*, b*) color space [22]. At the same time, pH was measured using the same pH meter described above. Drip and cooking loss were assessed subsequently on the same meat samples according to the method described by Honikel [23]. Briefly, for the drip loss, a 2 × 3 × 3 cm sample (approximately 80–100 g) was cut, put on top of a plastic mesh, and stored for 48 h in a sealed container at chill temperature (+5 °C). For cooking loss, each muscle chop was put in a plastic bag to avoid direct contact with water and cooked in a water bath until reaching the final core temperature of 75 °C. Warner–Bratzler Shear Force (WBSF) was measured as previously described [23] on six cores (diameter 1.13 cm) from each cooked meat chop using an Instron Universal Machine (model 1011, Instron Ltd., Wycombe, UK) fitted with a Warner–Bratzler (WB) device at a cross-head speed of 200 mm/min. WBSF is expressed as kg/cm^2^.

For the sensory analysis, muscle sections were transferred to the laboratories at the Research Centre on Animal Production (Reggio Emilia, Italy), where they were divided into 1.5 cm slices which were immediately vacuum-packed and stored at −20 °C until analysis, which was carried out within one month. The sensory analysis was then carried out by a panel of nine trained assessors according to the method described by Della Casa et al. [24]. The assessors were selected and trained according to the UNI EN ISO 8586:2012 [25]. The preparation of the test and the evaluation of the sensory quality of the products was operated according to the UNI EN ISO 13299:2010 “Sensory analysis—Methodology—General guidance for establishing a sensory profile” [26]. The analysis was carried out in a controlled environment (laboratory “CRPA Lab”), according to UNI EN ISO 8589:2014 [27]. Factors were evaluated with the use of a structured continuous scale of values between 1 and 10 (1 = absence of sensation, 10 = greatest intensity of sensation). Lean color and marbling (visible intramuscular fat) were visually assessed on raw samples. For these two parameters, a reference photographic scale (the same described by Dalla Casa et al. [24]) was provided to the assessors to help them estimate the surface characteristics. After the visual assessment, the samples were cooked on a hot plate until slices reached the core temperature of 70°. Sensory traits assessed on the cooked samples were: initial tenderness (effort required to cut the meat sample with incisor teeth), chewing tenderness (effort required to chew the meat sample with molar teeth), juiciness, final residue (presence of muscle fibers in the mouth at the end of chewing), chewiness (number of chews required to make the sample suitable for swallowing), aroma intensity, buttery aroma, and off-flavors (rancid, metallic, blood taste, etc.).

### 2.6. Statistical Analysis

All data were analyzed using the software JMP v14.3 (SAS Institute Inc., Cary, NC, USA). After logarithmic transformation, cortisol and CK values within the corresponding shipment were used in k-means cluster analysis in order to differentiate more or less stressful shipments. Models with 2, 3, and 4 clusters were tested, but the 2-cluster model was chosen, based on its higher fit statistics (Cubic Clustering Criterion (CCC) values of −2.2212, −2.5827, and −3.7648 for the 2-, 3-, and 4-cluster models, respectively). The 2-cluster model resulted in two groups, including 21 and 23 shipments (a more detailed description of the clusters will be given in the Results and Discussion section). For numerical variables, a linear mixed-model procedure was carried out with the aim of identifying statistical differences between the two clusters in the measures taken on departure, during transport, and at slaughter. Each shipment from the farm of origin was considered as an experimental unit and used as a random variable for all analyses. For blood parameters and meat quality, each shipment was used as the experimental unit, and the 10 measures for each shipment were considered as repeated measures with an autoregressive covariance structure (AR1). The cluster grouping was used as a fixed effect within the model. Means were separated on the basis of the least-square mean, and all pairwise multiple comparisons were performed using Tukey as a post-hoc test. For categorical variables, a nominal logistic model with the chi-square likelihood ratio test was carried out. A *p*-value ≤ 0.10 was considered as a tendency, a *p* ≤ 0.05 was considered statistically significant.

## 3. Results and Discussion

### 3.1. General Characteristics of the Shipments

The descriptive statistics of the shipments is reported in Table 1. Both the average TSWI and the scores obtained in the separate checklists were positive, with the only exception of some negative scores (minimum value: −1.75 pts) recorded in a few visited farms. Overall, the three average scores (1.75, 3.28, and 28.8 pts, for the farm, transport, and slaughter scores, respectively) were in the moderate-to-high range of the checklist (ranges: from −30.5 to 15.5 pts for the on-departure parameters, −18 to +9.5 pts for the transport, and −41.5 to +50.5 pts for the slaughter checklist, as described in the Material and Methods section). This indicates the absence of critically negative situations for the welfare of animals due to the fact that all practices observed in the present study were respectful of the European legislation on the protection of pigs during rearing, transport, and slaughter [16,17,18]. The total journey duration was low (on average 136 min), which may result from the proximity of the farms to the slaughterhouse (12 to 50 km) and the short waiting time of the truck at loading and before unloading (below 20 min on average, for both). Short travel durations are not uncommon in Northern Italy. Previous, larger retrospective studies carried out in the same area revealed that approximately one-half of the transports lasted below 90 min [15] and that the large majority of pigs (approximately 90%) were transported for less than 2 h [28]. The average lairage duration (9 ± 8.6 h) recorded in the present study resulted from the variable lairage times applied at the slaughterhouse audited in this study according to the arrival order (morning or later hours of the slaughter day). In particular, it was observed that some loads (26 shipments, corresponding to 59% of loads) were slaughtered within few hours after unloading, of which one was slaughtered immediately after unloading (minimum lairage duration of 3 min), whereas others (18 shipments, corresponding to 41% of loads) were kept in lairage overnight (lairage duration above 10 h). Although there is a certain degree of contradiction among studies about the definition of optimal lairage duration in relation to its effects on meat quality [29], a lairage of 2–3 h is generally recommended [4]. It has been observed that, in the case of heavy pigs, overnight lairage may increase the risk of pre-slaughter animal losses [15], although this effect seems to be largely dependent on the characteristics of the slaughterhouse such as stocking density, presence of large open windows, and use of sprinklers as cooling devices [28]. The number of pigs loaded per hour also presented a high variation (SD = 62), which likely reflects differences in farm and truck design (loading facilities), personnel training/experience, as well as handling techniques. Lastly, accidents during handling (such as slipping, falling, and overlaps) were observed on approximately a quarter of the animals (24% of animals observed having accidents at loading or at unloading). Such percentages are either considerably lower or slightly higher than those reported in other studies at loading and unloading [30,31,32].

### 3.2. Clusters Based on Blood Parameters

**Figure 1:**
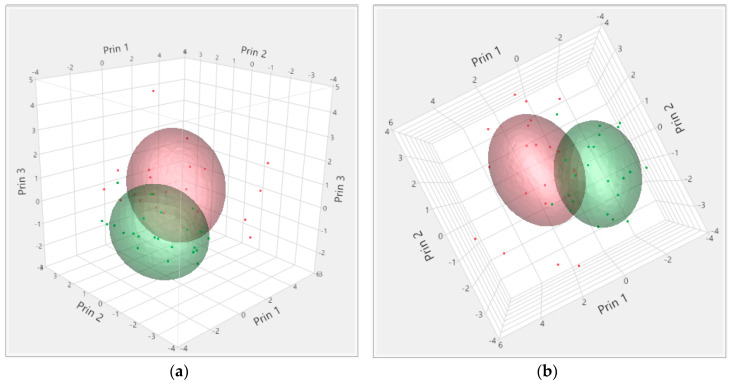
Three-dimensional representation of the shipments clustered according to blood cortisol and creatine kinase (CK) levels and seen from different angles (**a**,**b**). Each dot on the graph represents a shipment. Cluster 1 (red color, 21 shipments), Cluster 2 (green color, 23 shipments). The colored area represents the area around the cluster centroid.

Blood parameters allowed the separation of the shipments in two clusters (Figure 1). Cluster 1 (red color) included 21 shipments, and cluster 2 (green color) included 23 shipments. The average values of the two blood parameters (cortisol and CK) in the two clusters are summarized in Table 2. Cluster 1 included shipments with animals presenting higher average cortisol and CK levels than Cluster 2, therefore the two clusters were renamed Higher Stress (HS), and Lower Stress (LS) clusters, respectively. In this study, blood cortisol and CK levels were similar to those observed in lighter pigs in other studies [9,12,30,33,34], which may indicate the lack of difference in the physiological response to pre-slaughter stress between heavier and lighter pigs.

### 3.3. Differences in Transport Variables between Clusters

Table 3 presents the differences observed in a selected list of variables measured in the TSWI checklists between the two clusters. Variables were sorted by significance level (from higher to lower) in order to show first those having the highest significant effect.

Parameters significantly or tendentially differing between the two clusters were average vehicle speed during transport, slaughter score, TSWI, distance travelled, group stability, and pig behavior during handling at unloading. Overall, the observed differences between clusters in the parameters listed were in line with the expected differences between two groups of animals being subjected to different stress levels. More specifically, slaughter scores and TSWIs were higher in the LS than in the HS group (*p* = 0.01 and *p* = 0.04, respectively). The same was true also for average vehicle speed during transport and distance travelled (*p* = 0.002 and *p* = 0.05, respectively), which confirms the low impact of long-distance hauls when pigs are transported in good conditions (e.g., use of highway instead of secondary, country roads). Group stability tended to be higher (*p* = 0.07) in LS shipments, implying that during these shipments, the groups were less mixed (either at loading or during lairage), and less hierarchical fighting occurred. Irregular behaviors during handling at unloading (slipping, falling, and overlapping) tended to be higher (*p* = 0.07) in the HS shipments.

With respect to average vehicle speed during transport and distance travelled, as mentioned above, the LS cluster included shipments having a slightly longer (*p* = 0.05) travel distance but a notably higher (*p* = 0.002) average vehicle speed during transport. Considering that the average travel duration of all transports was relatively low (below 40 min in both clusters), it is likely that most transports for the LS cluster were carried out on smoother roads (e.g., on motorways or highways) instead of on rougher roads (e.g., country or city roads). Rougher travels, despite being shorter in duration, may have caused a higher stress response. The combination of travelling on a poor road surface and short transport might actually be even more stressful, as pigs mostly stand during the first phases of transport and so are more prone to injuries and falls [1,35]. Similar effects of the road type on the stress response and behavior were previously observed both in lambs and in pigs [36,37].

Regarding the calculated indexes, we observed that both the slaughter score and the TSWI were significantly higher in the LS than in the HS cluster (*p* = 0.01 and *p* = 0.04, respectively), indicating an overall better welfare both during the entire pre-slaughter period (from loading to stunning) and, in particular, at the slaughter plant. This indicates that the TSWI, in its current formulation, seems to be a valid tool to be used as a pre-slaughter stress indicator. However, it should also be considered that the absence of differences between clusters in the transport and farm scores may be due to the possibility that cortisol (and, to a lower extent, creatine kinase) is affected also by the acute stress response at slaughter, not only by the on-farm and transport stress response. Among the components of the TSWI, the slaughter score significantly differed between the two clusters (*p* = 0.01), whereas the differences in the transport and farm scores failed to reach statistical significance (*p* > 0.10). This result was quite surprising considering the variation in loading and departure conditions at the farms and in transport conditions, while the slaughterhouse conditions were always the same. The low SD of the slaughter score (Table 1) is therefore the result of the variation in those farm and transport variables which were also analyzed separately (e.g., unloading duration, lairage duration, pig behaviors during handling at unloading). Despite the limited variability in the slaughter score observed in the present study, both the slaughter score and the TSWI index seem to be sensible enough to identify stressful events occurring at slaughter or during the entire pre-slaughter period. However, a validation of the efficiency of the TSWI under more variable slaughter conditions is needed.

The limited variability in farm and transport scores may have had a masking effect, excluding the possibility to highlight differences in these indexes between clusters. Such a low variability might be due to the fact that all transports were carried out in full accordance with the EU legislation, allowing to obtain a moderate-to-high animal welfare score. These observations highlight the need to give a different weight to the parameters within the farm and the transport scores, increasing the importance of those parameters (i.e., average vehicle speed during transport, distance travelled, group stability, pig behaviors during handling, and loading duration) which, when considered separately, proved to have a more consistent effect on cluster differentiation. However, it should also be considered that monitoring a shorter list of pre-slaughter variables (either by summarizing them in a simplified index or by considering them separately) might lead to a more accurate prediction of the stress experienced by pigs during pre-slaughter handling.

The greater group stability observed in the LS cluster compared with the HS one (*p* = 0.07) confirms the effects of mixing unfamiliar pigs on increased aggression, poorer welfare, and increased blood CK [38] and salivary cortisol levels [39] compared with keeping pigs in their social groups, even when groups of pigs are mixed only at loading.

Overall, (1) combining the measure of two objective blood parameters (cortisol and CK) at slaughter may help use the physiological response of pigs to differentiate the impact of different transport systems on animal welfare; (2) the variables which differed more largely between the two clusters might be considered, under our conditions, the best descriptors of the welfare conditions experienced by Italian heavy pigs during pre-slaughter handling.

### 3.4. Differences in Meat Quality Traits and Serum Aldolase between Clusters

Table 4 summarizes the differences in meat quality and in blood aldolase levels between the two clusters. None of the instrumentally assessed meat quality parameters differed (*p* > 0.10). However, similarly to cortisol and CK, blood aldolase level was higher (*p* < 0.001) in the HS cluster compared to the LS one. This result may indicate a higher degree of muscle damage in the HS cluster pigs, which may be associated to the factors characterizing the HS cluster. All these characteristics result in acute stress and fatigue [1,4,40]. For example, long journeys, group mixing and accidents during handling may cause both direct (traumatic) and indirect (fatigue-induced) muscle damage (therefore leading to an increase in serum leaking enzymes), together with an increase in the stress response. Similarly, low slaughter scores are the consequence of a combination of unfavorable conditions occurring at the slaughter plant (unloading, lairage and stunning facilities, moving devices, etc.); therefore, they result in a similar effect. In an exploratory statistical model, shipments were clusterized based on the three blood parameters here assessed (cortisol, aldolase, and CK). Interestingly, with the only exception of one shipment, using three blood parameters resulted in no differences in shipments clusterization compared to the results which are presented in this study (data not shown). However, the model using also aldolase was discarded on the basis of the relatively limited literature available on aldolase compared to cortisol and CK, and aldolase was therefore used as a response variable. However, our data would support the use of blood aldolase as a long-term indicator of muscle damage in response to stress in future studies.

The juiciness score of the cooked meat was lower in the HS group despite the similar pH, drip loss and cooking loss values, and marbling scores observed between clusters, which in theory should lead to comparable sensory profiles [41]. This lack of difference in these technological meat quality traits makes it hard to explain the differences observed in the sensory parameters as a consequence of the different stress level to which the two clusters were exposed. It is however possible that the differences observed in these sensory traits might be due to marbling which, despite not reaching statistical significance (*p* = 0.3), was higher in the LS than in the HS group, therefore possibly determining an increase in the perceived juiciness and aromatic profile. However, it should also be considered that all the differences observed, although significant, were quite low (between 0.2 and 0.45 points differences on a 10-point sensory scale) and may not be perceived by the average consumer. Lastly, the overall sensory differences observed seem to indicate a more favorable sensory profile for the LS meat (higher juiciness, aroma intensity, and buttery aroma), except for the off-flavor score which, however, was lower than its standard level of acceptability (3 on a scale of 10 points).

Overall, the absence of differences in meat quality between clusters found in this study confirms the poor to moderate relationship between the physiological response to pre-slaughter stress and meat quality, as previously reported by a number of studies [12,13,14,42]. The moderate-to-high animal welfare experienced by pigs during transport in this study, resulting in a low meat quality variation, may explain this poor relationship. This observation is in agreement with the findings of a review on transport research, which concluded that when transport stress is mild, meat quality is not influenced significantly, as these effects are biased by rest time in lairage (overnight lairage in this study) [43].

## 4. Conclusions

This study was the first ever application of a new index (TSWI) as a possible pre-slaughter indicator to predict stress and meat quality in heavy pigs. This work allowed the identification, among the parameters included in the TSWI, of a list of pre-slaughter indicators which can be considered the best descriptors of the welfare conditions experienced by heavy pigs during pre-slaughter handling. Some of these indicators were simple transport variables (i.e., average vehicle speed during transport, distance travelled, group stability, and pig behaviors during handling at unloading), whereas others were complex welfare indexes (i.e., the welfare index at slaughter, and the overall transport and slaughter welfare index).

Although these first results appear promising, a further validation of the TSWI as a stress and meat quality predictor is needed under more variable pre-slaughter conditions. For a more reliable validation, future studies should assess:

(1) The correlations between individual blood and meat quality parameters and the presence of individual variation within the same load;
(2) The effects of the variables showing the largest difference between clusters on meat quality variation. To this end, it would also be of interest to assess the effect of the single behaviors observed during handling (slips, falls, and overlaps) on meat quality variation, as they are an expression of different physical and psychological condition of the pigs.

## Figures and Tables

**Table 1:** Descriptive statistics of the pig shipments (N = 44).

Parameter ^1^, U.M ^2^.	Average	S.D.	Min	Max
On-departure (at farm) score, pts	1.75	1.73	−1.5	5.5
Transport score, pts	3.28	1.64	0.5	6.5
Slaughter score, pts	28.08	2.27	24.5	31.0
TSWI ^3^ (farm + transport + slaughter), pts	33.11	3.48	26.0	42.0
Loading duration, min	46.7	21.5	20	110
Pigs loaded per hour, n	170	62	71	323
Waiting time at the farm before departure, min	13.7	5.0	3	25
Journey duration, min	37.2	18.8	18	90
Average speed during transport, km/h	73.3	16.4	42	111
Distance travelled, km	26.2	11.6	11	59
Ambient temperature, °C	16.8	8.5	−2.5	32
Waiting time at the slaughterhouse (before unloading), min	21.3	14.8	3	62
Total waiting time on the truck (farm + slaughterhouse), min	34.9	16.2	11	75
Unloading duration, min	16.9	5.1	9	30
Pigs unloaded per hour, n	433	125	200	870
Total journey duration (from loading to unloading), min	135.8	32.8	80	197
Lairage duration (from unloading to stunning), min	565.5	514.8	3	1393
Transport + lairage duration, min	701.3	519.0	159	1521
Behaviors (slipping, falling, overlapping):				
Loading, %	18.12	10.90	2.2	45.8
Unloading, %	5.43	3.55	0.0	16.33
Total, %	23.60	12.18	2.2	52.8

^1^ Detailed information on the variables assessed can be found in the Supplementary Materials; ^2^ Unit of Measurement; ^3^ Transport and Slaughter Welfare Index.

**Table 2:** Summary of the characteristics of the two clusters (backtransformed data are presented between square brackets).

Parameter, U.M. ^3^	Cluster 1 (HS ^1^) (N = 210 Pigs)		Cluster 2 (LS ^2^) (N = 230 Pigs)		*p*-Value
	Estimate	SE ^4^	Estimate	SE ^4^	-
log Cortisol, ng/mL	1.10 [12.72]	0.03	0.97 [9.32]	0.03	0.007
log CK, U/L	3.37 [2394]	0.02	3.20 [1583]	0.02	<0001

^1^ Higher Stress; ^2^ Lower Stress; ^3^ Unit of Measurement; ^4^ Standard Error.

**Table 3:** Differences in the measured transport and slaughter variables between the two clusters. Variables are sorted by significance level in decreasing order of significance (from higher to lower).

	HS ^1^		LS ^2^		
Number of Shipments	21		23		
Variable ^3^	Estimate	SE ^4^	Estimate	SE	*p*-Value
Average vehicle speed during transport, km/h	65.62	3.23	80.35	3.09	0.002
Slaughter score, pts	27.19	0.47	28.89	0.44	0.012
TSWI ^5^ (farm + transport + slaughter), pts	32.00	0.73	34.13	0.70	0.041
Distance travelled, km	22.62	2.45	29.48	2.34	0.050
Stable (unmixed) groups, odds ratio	0.48	0.32	0.74	0.32	0.072
Behaviors (slipping, falling, overlapping) at unloading, %	6.49	0.75	4.58	0.72	0.075
Total behaviors (slipping, falling, overlapping), %	26.50	2.62	20.94	2.50	0.132
Loading duration, min	51.48	4.63	42.30	4.43	0.160
Behaviors (slipping, falling, overlapping) at loading, %	20.03	2.37	16.38	2.27	0.272
Pigs loaded per hour, n	159.19	13.53	179.13	12.93	0.293
Total journey duration (from loading to unloading), min	140.81	7.16	131.17	6.84	0.336
Waiting time at the farm (before departure), min	14.29	1.10	13.13	1.05	0.450
On-departure (at farm) score, pts	1.57	0.38	1.91	0.36	0.519
Total waiting time on the truck (farm + slaughterhouse), min	36.10	3.58	33.87	3.42	0.655
Unloading duration, min	17.29	1.13	16.61	1.08	0.667
Journey duration, min	35.95	4.14	38.39	3.96	0.672
Waiting time at the slaughterhouse (before unloading), min	21.81	3.26	20.74	3.12	0.814
Transport score, pts	3.24	0.36	3.33	0.35	0.861
Pigs unloaded per hour, n	434.38	27.57	431.52	26.35	0.941
Transport + lairage duration min	705.48	114.60	697.52	109.50	0.960
Ambient temperature, °C	16.88	1.87	16.75	1.79	0.961
Lairage duration (from unloading to stunning), min	564.67	113.67	566.35	108.61	0.992

^1^ Higher Stress, ^2^ Lower Stress; ^3^ Detailed information on the variables assessed can be found in the Supplementary Materials; ^4^ Standard Error ^5^ Transport and Slaughter Welfare Index.

**Table 4:** Results of the linear mixed-model procedure to identify statistical differences between the two clusters in the measured meat quality parameters and in blood aldolase levels.

	HS ^1^		LS ^2^		
Number of Samples	210		230		
Variable	Estimate	SE ^3^	Estimate	SE	*p*-Value
log Aldolase, U/L	1.7106 [51.35]	0.031	1.5693	0.030 (37.10)	0.002
pH45	6.00	0.04	6.00	0.04	0.938
pH24	5.53	0.02	5.50	0.02	0.254
Drip Loss, %	1.16	0.07	1.03	0.06	0.180
L*	47.08	0.83	48.95	0.79	0.109
a*	4.20	0.24	4.01	0.23	0.572
b*	4.77	0.23	5.09	0.22	0.307
Hue	0.87	0.02	0.92	0.02	0.097
Chroma	6.46	0.31	6.62	0.29	0.697
Cooking loss, %	26.04	0.75	26.58	0.71	0.609
WBSF, kg/cm^2^	3.86	0.15	3.91	0.14	0.801
Color score	4.57	0.12	4.72	0.11	0.362
Marbling score	4.76	0.25	5.11	0.24	0.308
Initial tenderness	5.32	0.15	5.57	0.14	0.231
Chewing tenderness	4.86	0.14	5.11	0.13	0.212
Juiciness	3.58	0.14	4.09	0.14	0.021
Final residue	2.90	0.06	3.04	0.06	0.125
Chewiness	5.28	0.10	5.50	0.10	0.138
Aroma intensity	5.11	0.12	5.56	0.12	0.014
Buttery aroma	2.76	0.08	2.99	0.08	0.054
Off-flavours	2.18	0.05	2.38	0.05	0.008

^1^ Higher Stress, ^2^ Lower Stress; ^3^ Standard Error.

## Supplementary Materials

The following are available online at https://www.mdpi.com/2076-2615/10/6/945/s1.
